# Eukaryotic Elongation Factor 2 Kinase Activity Is Required for the Phenotypes of the *Rpl24*^*Bst*^ Mouse

**DOI:** 10.1016/j.jid.2022.06.019

**Published:** 2022-12

**Authors:** John R.P. Knight, Christopher G. Proud, Giovanna Mallucci, Tobias von der Haar, C. Mark Smales, Anne E. Willis, Owen J. Sansom

**Affiliations:** 1 Beatson Institute, Cancer Research UK, Glasgow, United Kingdom; 2 Division of Cancer Sciences, Faculty of Biology, Medicine and Health, The University of Manchester, Manchester, United Kingdom; 3 Lifelong Health, South Australian Health and Medical Research Institute, Adelaide, Australia; 4 Department of Biological Sciences, University of Adelaide, Adelaide, Australia; 5 UK Dementia Research Institute at The University of Cambridge, Cambridge, United Kingdom; 6 Department of Clinical Neurosciences, School of Clinical Medicine, University of Cambridge, Cambridge, United Kingdom; 7 School of Biosciences, University of Kent, Canterbury, United Kingdom; 8 MRC Toxicology Unit, University of Cambridge, Cambridge, United Kingdom; 9 Institute of Cancer Sciences, University of Glasgow, Glasgow, United Kingdom

**Keywords:** eEF2K, eukaryotic elongation factor 2 kinase

To The Editor

The variant murine *Rpl24*^*Bst*^ allele reduces gene expression by 40% and results in a white belly spot and kinked tail of variable severity and penetrance (Oliver et al., 2004). White belly spots lack melanocytes owing to defects in their motility during development, whereas kinked tails result from fused or wedge-shaped vertebrae (Oliver et al., 2004). *Rpl24* encodes the protein RPL24, which is incorporated into the ribosome in the cytoplasm. Notably, altered expression of five other ribosomal protein genes—*Rps7, Rps21, Rps19, Rps20*, and *RACK1*—result in belly spots (Dinh et al., 2021; McGowan et al., 2008; Volta et al., 2013; Watkins-Chow et al., 2013). Despite this genetic evidence of a link between ribosomal proteins and belly spots, the mechanism(s) behind the phenotype are unknown. The *Rpl24*^*Bst*^ mutation reduces protein synthesis in embryonic neural tube cells (Kondrashov et al., 2011) and models of cancer (e.g., as reported in Knight et al. [2021]), suggesting that reduced protein synthesis underpins the developmental defects. In agreement, reduced RACK1 limited protein synthesis in murine embryonic fibroblasts (Volta et al., 2013). However, the other belly-spot variants do not affect protein synthesis (Kondrashov et al., 2011; Watkins-Chow et al., 2013). Thus, to understand the mechanistic link between protein synthesis and belly spots, experiments are required to directly alter protein synthesis rates in variant mice.

We previously showed that *Rpl24*^*Bst*^ mutation suppresses protein synthesis through the activation of eukaryotic elongation factor 2 kinase (eEF2K) (Knight et al., 2021). Suppression of protein synthesis in *Rpl24*^*Bst*^ variants is completely reversed by eEF2K inactivation, providing a tool to reverse the protein synthesis defects in *Rpl24*^*Bst*^ variants. The *Eef2k*^*D273A*^ allele is a germline knockin that dramatically reduces eEF2K catalytic activity throughout the whole mouse from conception (Gildish et al., 2012). We therefore used the *Eef2k*^*D273A*^ allele to assess the influence of eEF2K inactivation on *Rpl24*^*Bst*^ phenotypes. To achieve this, belly spot and tail severity were scored using a scale from 0 (normal) to 4 (severe) for mice generated in our previous study (Supplementary Figure S1a). The combined belly-spot and tail score of *Rpl24*^*Bst/+*^ mice has a median of 3 of 8 (Figure 1a). In comparison, the belly-spot and tail score of *Rpl24*^*Bst/+*^ *Eef2k*^*D273A/D273A*^ mice was significantly lower at only 1.5. Furthermore, there is a lower incidence of severe phenotypes, with only 7% of *Rpl24*^*Bst/+*^ *Eef2k*^*D273A/D273A*^ mice scoring ≥5 compared with 34% of *Rpl24*^*Bst/+*^ mice. Therefore, inactivation of eEF2K suppresses the observable skin and tail phenotypes of the *Rpl24*^*Bst*^ mutation. The main contributing factor to this difference is the tail score, with 35% of tails scoring >2 in *Rpl24*^*Bst/+*^ mice compared with 3.6% in *Rpl24*^*Bst/+*^ *Eef2k*^*D273A/D273A*^ mice (Supplementary Figure S1b).

**Figure 1:**
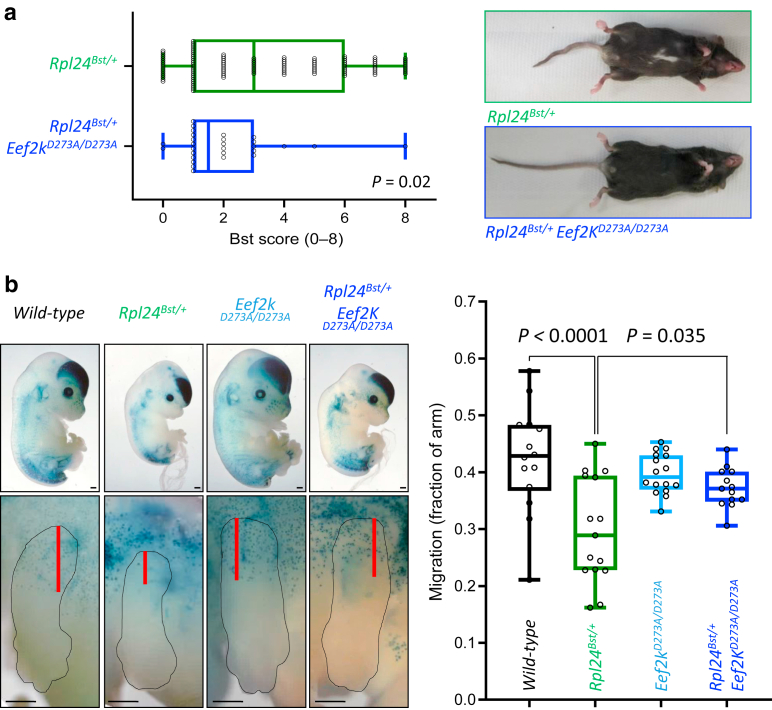
**Inactivation of eEF2K reverses the phenotypes of the *Rpl24*^*Bst*^ mutation.** (**a**) Quantification of Bst score from mice with *Rpl24*^*Bst*^ mutation alone (n = 115 mice) or in combination with *Eef2k*^*D273A/D273A*^ (n = 28 mice). Significance was determined by Kolmogorov‒Smirnov test. Right: representative (median Bst score) image of *Rpl24*^*Bst/+*^ and *Rpl24*^*Bst/+*^*Eef2k*^*D273A/D273A*^ mice. (**b**) Images of transgenic *Dct-lacZ* mice at E13.5 mice stained for β-galactosidase (blue) within melanocytes. The upper image shows whole embryos, and the lower image shows an expanded view of one forelimb. Bars = 500 μm. Right: quantification of melanocyte migration scored as the distance from torso to furthest melanocyte/length of forelimb. Significance was determined by one-way ANOVA (Tukey’s multiple comparison). Left to right n = 14, 15, 16, and 13. For both graphs, boxes mark the 25th and 75th percentiles, and the central lines mark the median. Each point represents an individual mouse/embryo. Bst, belly-spot and tail; E13.5, embryonic day 13.5; eEF2K, eEF2K, eukaryotic elongation factor 2 kinase.

Next, we took a complementary approach measuring the effect of the *Rpl24*^*Bst*^ and *Eef2k*^*D273A*^ mutations on melanocytes using the *Dct-lacZ* system (Mackenzie et al., 1997). Genetically engineered *Dct-lacZ* mice express β-galactosidase from the melanocyte-specific dopachrome tautomerase (*Dct)* promoter, allowing whole-mount visualization and quantification of melanocyte location. On embryonic day 13.5, melanocytes are transiting the forelimbs of embryos, with the fraction of melanocyte-positive forelimb a metric for changes in melanocyte migration. We observe a significant reduction in melanocyte migration in *Rpl24*^*Bst/+*^ embryos, consistent with their belly spots in adulthood, whereas the inactivation of eEF2K has no effect (Figure 1b). Compared with that in *Rpl24*^*Bst/+*^ embryos, melanocyte migration is significantly reverted in *Rpl24*^*Bst/+*^ *Eef2k*^*D273A/D273A*^ embryos (Figure 1b). Thus, eEF2K activity is required for the perturbed melanocyte migration phenotype of *Rpl24*^*Bst*^ embryos. Why the reverted migration in *Rpl24*^*Bst/+*^ *Eef2k*^*D273A/D273A*^ embryos (Figure 1b) does not correlate with a reversal of adult belly spot phenotype (Supplementary Figure S1b) is unclear. It is possible that migration is reversed at embryonic day 13.5 but subsequently slows to produce a belly spot. Unfortunately, staining of *Dct-lacZ* is not possible at later embryonic stages owing to reduced skin permeabilization (Mackenzie et al., 1997).

We noted that *Rpl24*^*Bst/+*^ and *Rpl24*^*Bst/+*^ *Eef2k*^*D273A/D273A*^ mice were weaned at lower than Mendelian frequencies: *Rpl24*^*Bst/+*^ mice at 1 in 3 (32.1%) when expected at 1 in 2 and *Rpl24*^*Bst/+*^ *Eef2k*^*D273A/D273A*^ mice even less frequently at 1 in 5 (20.7%) (Figure 2a). Unexpectedly, when we compared the frequency of *Rpl24*^*Bst/+*^ *Eef2k*^*D273A/D273A*^ mice with the experimentally determined frequency of *Rpl24*^*Bst/+*^ mice (32.1%), we found this to be significantly different (Figure 2a). Thus, the viability of *Rpl24*^*Bst/+*^ mice is at least in part dependent on eEF2K activity. To analyze this effect further, we calculated the frequencies of the same genotypes in embryonic day 13.5 embryos, finding that the incidence of the *Rpl24*^*Bst*^ mutation was close to Mendelian with active or inactive eEF2K (Figure 2b). From this, we conclude that the reduced viability of *Rpl24*^*Bst/+*^ *Eef2k*^*D273A/D273A*^ mice occurs between embryonic day 13.5 and weaning. Therefore, despite being responsible for the skin and tail phenotypes of the *Rpl24*^*Bst*^ mutation, eEF2K promotes the preweaning survival of *Rpl24*^*Bst*^-variant mice. Embryo viability could not be determined during our analysis. Thus, *Rpl24*^*Bst/+*^ *Eef2k*^*D273A/D273A*^ embryos with the greatest reversion in belly-spot phenotype may not survive, potentially explaining the observed discrepancy in melanocyte migration and adult belly spots in this genotype. These data provide substantive evidence of a mechanistic link between RPL24 and eEF2K at an organism level.

**Figure 2:**
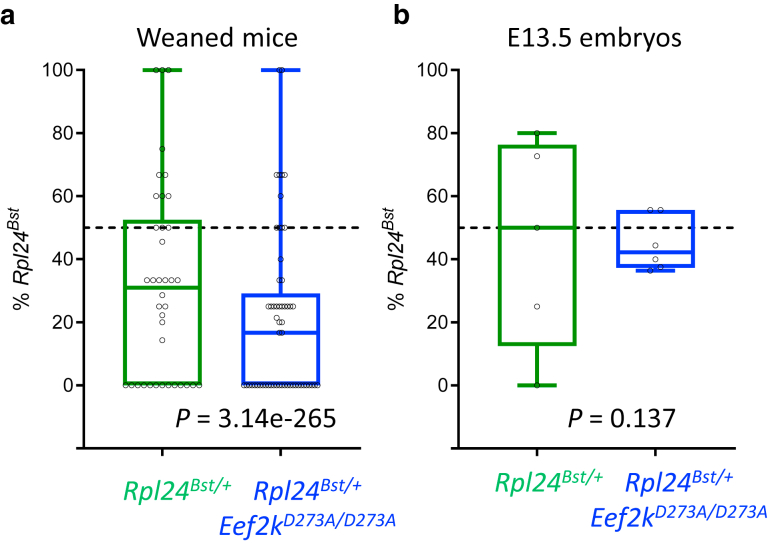
**eEF2K is required for preweaning survival of *Rpl24*^*Bst/+*^mice.** (**a**) The percentage of *Rpl24*^*Bst/+*^ mice weaned per litter is plotted in black, with each point representing an individual litter. The box marks the 25th and 75th percentiles, and the central line marks the median incidence of *Rpl24*^*Bst*^ mutation. Significance was assessed by chi-square test using the average incidence of *Rpl24*^*Bst/+*^ mice (n = 38 litters) as the expected frequency compared to the actual frequency of *Rpl24*^*Bst/+*^*Eef2k*^*D273A/D273A*^ mice (n = 57 litters). (**b**) Analysis as in (a) but for embryos at E13.5. For *Rpl24*^*Bst/+*^ embryos n = 5 litters and for *Rpl24*^*Bst/+*^*Eef2k*^*D273A/D273A*^ embryos n = 6. E13.5, embryonic day 13.5; eEF2K, eukaryotic elongation factor 2 kinase.

Similar to eEF2K inactivation, deletion of *p53* in *Rpl24*^*Bst*^ variants reversed belly-spot and tail defects while also reducing the ratio of *Rpl24*^*Bst/+*^ mice (Barkić et al., 2009). *Rpl24*^*Bst/+*^ *p53*^*‒/‒*^ mice showed reduced embryonic apoptosis and dependence on p21 for viability. Suggesting a shared mechanism, eEF2K activity is required for apoptosis at various stages of development and promotes p21 expression (Chu et al., 2014; Liao et al., 2016). In addition, ribosome biogenesis stress activates both p53 and eEF2K (Gismondi et al., 2014; Knight et al., 2016). Interestingly, the belly-spot phenotypes of *Rps7*, *RACK1*, *Rps19*, and *Rps20* mutations were also rescued by deletion of *p53* (Dinh et al., 2021; McGowan et al., 2008; Watkins-Chow et al., 2013). Whether inactivation of eEF2K would also rescue these belly-spot phenotypes should now be determined

This work extends the relationship between RPL24 and eEF2K to include melanocyte migration, tail deformation, and organism survival. *Rpl24*^*Bst/+*^ mice have been used in cancer studies, to model retinal degeneration, and to study brain development (Herrlinger et al., 2019; Riazifar et al., 2015). This work shows that eEF2K may play a role across these diverse biological settings, with further work merited to investigate its importance.

### Declaration for animal use

Studies were carried out under license from the UK Home Office (60/4183 and 70/8646). Mice were maintained in open-top cages with a 12-hour light/dark cycle and free access to water and diet. Experiments were initiated on inbred C57BL/6J male and female mice aged between 6 and 12 weeks, without blinding or randomization.

### Data availability statement

No datasets were generated or analyzed during this study.

## ORCIDs

John R. P. Knight: http://orcid.org/0000-0002-8771-5484

Christopher G. Proud: http://orcid.org/0000-0003-0704-6442

Giovanna Mallucci: http://orcid.org/0000-0001-8504-1191

Tobias von der Haar: http://orcid.org/0000-0002-6031-9254

C. Mark Smales: http://orcid.org/0000-0002-2762-4724

Anne E. Willis: http://orcid.org/0000-0002-1470-8531

Owen J. Sansom: http://orcid.org/0000-0001-9540-3010

## **Conflictof Interest**

OJS reports funding unrelated to work in this project from Astra Zeneca, Novartis, RedX, and Cancer Research Horizons. The remaining authors state no conflict of interest.

### Disclaimer

The funders had no role in study design, data collection, and interpretation or in the decision to submit the work for publication.

## Author Contributions

Conceptualization: JRPK, OJS; Formal Analysis: Funding Acquisition: GM, TvdH, CMS, AEW, OJS; JRPK; Investigation: JRPK; Methodology: JRPK; Project Administration: JRPK, OJS; Resources: CGP; Supervision: OJS; Writing - Original Draft Preparation: JRPK; Writing - Review and Editing: JRPK, CGP, GM, TvdH, CMS, AEW, OJS

## Figures and Tables

**Supplementary Figure S1:**
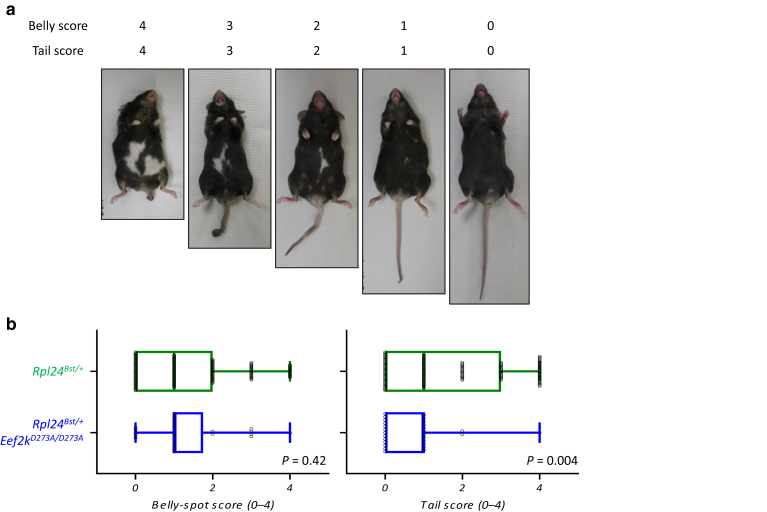
**The spectrum of belly-spot and tail scores.** (**a**) Examples of mice exhibiting each score on the belly-spot and tail scale. These mice show equivalent scores for each parameter for ease of representation, but equivalent scores between both metrics were not always the case. (**b**) Graphs showing the belly-spot and the tail scores that comprise the overall belly-spot and tail score shown in Figure 1a. *Rpl24*^*Bst/+*^ is shown in green; n = 115 mice. *Rpl24*^*Bst/+*^*Eef2k*^*D273A/D273A*^ is shown in blue; n = 28 mice. The box marks the 25th and 75th percentiles, and the central line marks the median. Each point represents an individual mouse. Significance was tested by Kolmogorov‒Smirnov test.

## Supplementary Materials and Methods

### Materials availability

All materials are freely available, where legally permitted, on acceptance of a material transfer agreement.

### Belly-spot and tail scoring

Genotyping was carried out by Transnetyx (Memphis, TN). Scoring was carried out at the endpoint of published experiments that determined sample size, with no power analysis performed for this study. Scoring was performed regardless of the presence of other alleles such as *Villin*^*CreER*^, *Apc*^*fl*^, *Apc*^*Min*^, or *Kras*^*G12D*^ because these alleles do not manifest a belly-spot or tail phenotype.

### Dct-lacZ

Overnight vaginal plugs were designated embryonic day 0.5, and embryos were collected on embryonic day 13.5. Embryo studies were performed blind. Tails were used for genotyping, and the remainder were fixed in ice-cold 0.25% glutaraldehyde (G6257, Sigma-Aldrich, St. Louis, MO) in PBS, on a rotational mixer for 30 minutes at 4 °C. Embryos were washed in cold PBS and permeabilized in two rounds of 2 mM magnesium chloride, 0.1% sodium deoxycholate (D6750, Sigma-Aldrich), and 0.02% NP40 (74385, Sigma-Aldrich) in PBS for a total of 40 minutes at room temperature. Embryos were stained in 2 mM magnesium chloride, 0.1% sodium deoxycholate, 0.02% NP40, 4.7 mM potassium hexacyanoferrate (II) trihydrate (P9387, Sigma-Aldrich), 4.8 mM potassium hexacyanoferrate (III) (P8131, Sigma-Aldrich), and 0.5 mg/ml X-Gal (V394A, Promega, Madison, WI) in PBS at 4 °C on a rotational mixer protected from light for 36 hours. Embryos were washed in PBS, visualized by light microscopy, and stored in formalin. The sample size required for significance was estimated on the basis of pilot studies using G∗Power (Faul et al., 2009).
